# Expression of TLR4-MyD88 and NF-*κ*B in the Iris during Endotoxin-Induced Uveitis

**DOI:** 10.1155/2010/748218

**Published:** 2010-08-03

**Authors:** Shang Li, Hong Lu, Xiaofeng Hu, Wei Chen, Yingzhi Xu, Jing Wang

**Affiliations:** ^1^Departmnet of Ophthalmology, Beijing Chaoyang Hospital, Capital Medical University, No. 8 Baijiazhuang Road, Chaoyang District, Beijing 100020, China; ^2^Department of Ophthalmology, Haidian Maternal and Child Health Hospital, Tonji Medical University, Beijing 100080, China

## Abstract

*Purpose*. To observe the expression of Toll-like receptor-4 (TLR4), myeloid differentiation factor 88 (MyD88), and nuclear factor kappa B p65 (NF-*κ*B p65) in iris tissue during endotoxin-induced uveitis (EIU) and evaluate the significance of these factors in uveitis. *Methods*. Wistar rats were randomly divided into 5 groups (0 h, 12 h, 24 h, 48 h, and 72 h, n = 10/group). Animal model of acute anterior uveitis was established by a hind footpad injection of 200 *μ*g Cholera vibrio LPS. Expression of TLR4, MyD88, and NF-*κ*B p65 in iris ciliary body tissue was detected through immunohistochemical staining. *Results*. Expression of TLR4 was not detected in normal iris-ciliary body complex, TLR4 positive cells with round morphology appeared in the iris stroma 12 hours after injection, significantly increased (*P* < .001) 48 hours after injection, and decreased gradually 72 hours after injection. Expression of MyD88 and NF-*κ*B p65 is consistent with the change of the TLR4. *Conclusions*. The increased expression of TLR4 and its downstream signal transduction moleculesMyD88, NF-*κ*B p65 indicate the potential role of pathway in the pathogenesis of acute anterior uveitis (AAU).

## 1. Introduction

The Toll-like receptors (TLRs) are a recently discovered family of innate immune recognition receptors (PRRs). Innate immune system is rapidly activated by recognition of pathogenic microorganisms and special structure of cell wall that is a highly conserved pathogen-associated molecular patterns (PAMPs) [1]. There are at least 13 human TLRs identified to date, in which Toll-like receptor 4 (TLR4) is the earliest discovered and the most studied. The main function of TLR4 is to identify the lipopolysaccharide (LPS) of Gram-negative bacterial cell wall and activates innate immune system. Chen et al. recently [2] reported that TLR4 was highly expressed in the surface of iris-ciliary macrophages in the rats with uveitis induced by footpad injection of Vibrio cholera lipopolysaccharide. It suggested that TLR4 may be involved in the pathogenesis of acute anterior uveitis (AAU). In our study, changes in the expression of TLR4, downstream transduction molecules MyD88, and NF-*κ*B were investigated in iris-ciliary during EIU by the same research method. The role of TLR4 transduction pathway was further explored in in the pathogenesis of AAU.

## 2. Materials and Methods

### 2.1. Material

#### 2.1.1. Animal

Adult male pathogen-free Wistar rats (8–10 weeks old, weighing 150–200 g) were obtained from Vital River Laboratory Animal Technology Co. Ltd (Beijing, China). Fifty animals were used in the study. Animals were randomly divided into five groups (*n* = 10 per group) for the following time points: before LPS injection (0 h; control group) and 6 h, 12 h, 24 h, and 48 h after LPS injection.

#### 2.1.2. Reagents

Lipopolysaccharide (V. cholera, classical Biotype, serotype Ogawa) was kindly provided by Lanzhou Institute of Biologic Products (Lanzhou, China). Rabbit Polyclonal antibody TLR4 and MyD88, mouse monoclonal antibody NF-*κ*B p65 was purchased from Santa Cruz Biotechnology (Santa Cruz, CA). Vectastain ABC-peroxidase kits were purchased from Vector Laboratory (U.S.A). Affinity purified normal rabbit IgG was purchased from Boster Biotechnology (Wuhan, China). Mouse IgG1 was purchased from Serotec (Oxford, UK), DAB kit was purchased from Zhongshan Goldbridge Biotechnology (Beijing, China).

#### 2.1.3. Instruments and Equipment

 Removal of the iris was under a stereo microscope (Leica-M165 C; Leica, Wetzlar, Germany). Slides were examined under an optical microscope (Leica-DM-4000B; Leica, Wetzlar, Germany). Images were captured by a slit lamp with an anterior segment camera system (Topcon SL-D7; Topcon, Japan), and analysis using image-management software (Adobe Photoshop CS3. 10.0; Adobe Systems, Mountain View, CA).

### 2.2. Methods

#### 2.2.1. Animal Model

Endotoxin-induced uveitis was induced as previously described [3]. Mice received a single injection of 200 *μ*g [4, 5] LPS dissolved in 100 *μ*L sterile saline (NS) in one rear footpad. The eyes were examined by slit microscope before the injection and at different time after the injection, and the intensity of anterior segment inflammation was evaluated by slit lamp.

#### 2.2.2. Inflammatory Scoring

The intensity of anterior segment inflammation was examined by slit microscope before the injection and at 2 h intervals after the injection. Inflammatory signs were recorded in detail, and photographs were taken. The severity of uveitis was graded from 0 to 4 by a investigator blinded to study protocol [6] as follows: 0 = no  inflammation; 1 = discrete vasodilatation of the iris and the conjunctiva vessels; 2 = moderate dilatation of the iris and the conjunctival vessels with moderate flare in the anterior chamber; 3 = intense iridal hyperemia with intense flare in the anterior chamber; and 4 = the  same clinical signs as 3 with fibrinous exudates in the pupillary area.

#### 2.2.3. Animal Perfusion and Specimen Preparation

 Intracardiac perfusion of rats was performed at different time points in order to eliminate the effect of blood to immunohistochemical staining. Animals were deeply anesthetized using 17.5% chloral hydrate (2 mL/kg**) **by intraperitoneal injection. Through an abdominal incision in the midsagittal plane to expose the chest wall, the rat was flushed through the left ventricle with 250–300 mL of phosphate-buffered saline (PBS), 1 IU heparin per mL of PBS until the outflow becomes colorless, then 4% paraformaldehyde(250 mL) was flushed through the heart. Movements in the limbs and tail (fixation has reached the extremities) indicated adequate perfusion [7]. After being fixed in 4% paraformaldehyde for additional 1-2 hs, the eyes were put into petri dishes filled with PBS. The iris-ciliary body complex was gently dissected into 3~4 segments under the stereomicroscope and stored in Eppendorf tube with PBS at −80°C.

#### 2.2.4. Histopathology

Rats were killed by overdose of pentobarbital (100 mg/kg) 24 hours after being immunized with LPS. The eyes of rats were enucleated and placed in 10% neutral buffered formalin solution for 24 h. After stationary liquid was washed out, tissue sample was immersed in 50%, 75%, 80%, 90%, and 100% alcohol for 1 h, respectively, to dehydrate. Then, the tissue was put into paraffin for 1 h × 3 for embedding after being treated with xylene for 30 minutes. Sagittal sections (4 *μ*m thick) were cut near the optic nerve head and stained with hematoxylin and eosin.

#### 2.2.5. Immunohistochemistry

 The prepared tissues were put into 24 well plate. After three rinses with PBS (5 min each), 0.3% triton-X 100 was used to perforate the cell membrane for 30 min. Then, endogenous peroxidase activity in the whole mounts was blocked by 0.3% H_2_O_2_ for 30 min at room temperature. After another three rinses with PBS, the tissues were blocked with 5% goat serum for 30 min, and incubated with primary antibody against TLR4, MyD88, and NF-*κ*B p65 (dilution, 1 : 50) overnight at 4°C. After several washes in PBS, the tissues were incubated with biotinylated antirabbit and antimouse secondary antibody (1 : 200) for 2 h at room temperature. After three further washes in PBS, the tissues were incubated with mixture of A and B (1 : 100) for 30 min at 37°C. Staining was visualized by the biotin-avidin-peroxidase method using diaminobenzidine as chromogen. Negative controls were performed by replacing the primary antibody with species-matched and isotype-matched antibodies with the same concentration of the primary antibody.

### 2.3. Quantitative Analysis

The method employed in our study involved counting the total number of immunopositive cells per iris segment (a strip 0.29 mm or 1 graticule in width from the pupil margin to iris base chosen randomly around the circumference). The mean length of the irides (base-pupil margin) in the present study was approximately 1 mm (0.97 mm or 3.4 graticule lengths); therefore, the mean area of a segment was 0.281 mm^2^. A minimum of two and up to five segments were counted per iris from one eye, and an overall mean density/mm^2^ per animal was obtained [8]. Cells were counted by a blinded investigator (one of the authors, who was unaware of the treatment). Different layers of stained cells could be distinguished in separate focal planes of the whole mounts. All cells of different layers in one field were counted under a microscope with 20X or 40X objective lens.

### 2.4. Statistical Analysis

Quantitative data were expressed as means ± standard deviation (SD) and were analyzed with one-way analysis of variance (ANOVA) followed by Significant Difference Procedure (LSD) test for multiple comparisons among experimental groups with control groups. Statistical analysis was performed using the SPSS 11.5 (SPSS Inc., Chicago, IL) statistical software. *P* value less than  .05 was considered statistically significant.

## 3. Result

### 3.1. Inflammatory Manifestation of EIU

**Figure 1:**
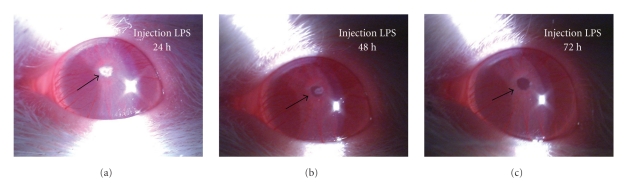
Clinical manifestation of EIU: (a) The image shows the eye 24 h after the LPS injection. Note the fibrinous pupillary membrane (arrow). (b) The image shows the eye 48 h after the LPS injection. The fibrinous pupillary membrane has not been all absorbed (arrow). (c) The image shows the eye 72 h after the LPS injection. The fibrinous pupillary membrane has been absorbed (arrow).

**Figure 2:**
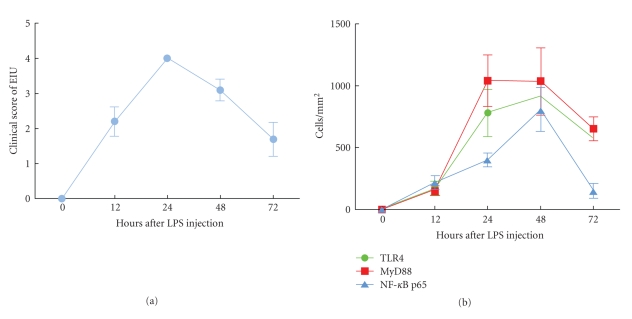
(a) The clinical score of EIU at different time (Data were expressed as means ± SD): the intensity of the anterior segment inflammation was evaluated at 0~12 h inflammation reached a maximum at 24 h inflammation subsided gradually after 24 h. (b) Expression of TLR4, MyD88 and NF-*κ*B p65 during iris at different time (Data were expressed as means ± SD): positive cells gradually increased at 0~24 h. Expression of MyD88 at 48 h compared with 24 h was no significant difference (*P* = .940). Expression of TLR4 and NF-*κ*B p65 at 48 h compared with 24 h was significant difference (*P* = .049, *P* = .000). Expression of TLR4 and NF-*κ*B p65 declined at 72 h.

 In addition to the control group, ocular inflammatory signs were observed in the each rat of remaining four groups after LPS injection. Conjunctival edema, ciliary congestion, and blood vessels dilatation in the iris began to appearance within 4~6 h after the LPS injection. At 12~16 h, aqueous flaring and fibrinous pupillary membrane was seen, reaching a maximum at 22~24 h. Occlusion of pupil even was found by slit microscope (Figure 1(a)). Generally, inflammation subsided gradually after 24 h, and the exudation had decreased 48 h after LPS injection (Figure 1(b)). At 72~76 h, fibrinous pupillary membrane had been completely absorbed, but only mild ciliary congestion remained. The score of EIU in Wistar rat at different time was described in Figure 2(a) and Table 1.

### 3.2. Histologic Changes

**Figure 3:**
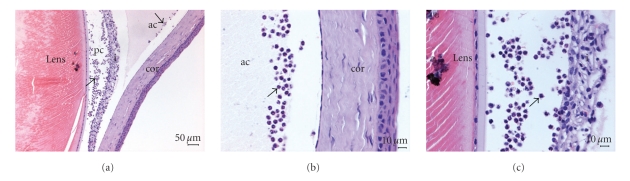
Histological changed after LPS immunized at 24 hours in Wistar rat (HE staining): (a) There were a lot of inflammatory cells in anterior and posterior chamber. Arrows indicated positive cells (Bar = 50 *μ*m). (b) There were a lot of inflammatory cells in anterior chamber. (c) There were a lot of inflammatory cells in posterior chamber (Bar = 10 *μ*m).

H-E staining results were consistent with inflammatory manifestations in Wistar rats at 24 h after LPS immunization. A large number of infiltration of inflammatory cells and fibrin exudations could be seen in the anterior and posterior chamber (Figure 3(a)); massive neutrophil adhered behind corneal endothelial cell (Figure 3(b)); thickened iris stroma with vasodilatation and a majority of inflammatory cell infiltration in the vitreous were observed (Figure 3(c)).

### 3.3. Expression of TLR4

**Figure 4:**
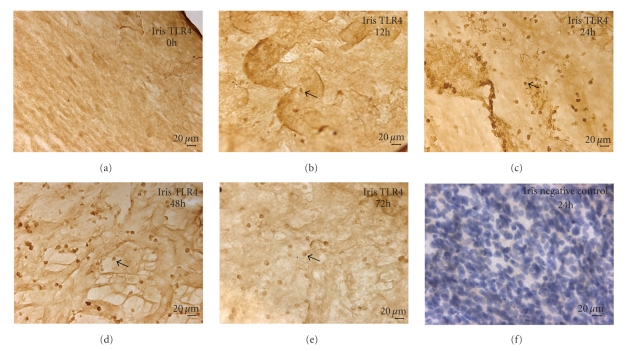
Immunohistochemistry of TLR4 (DAB): (a) TLR4 could not be detected in the iris at 0 h. (b) The TLR4 located adjacent to blood vessels in the iris at 12 h. (c) Most of TLR4 expressed in the iris at 24 h. (d) The TLR4 positive cells were shown at 48 h. (e) TLR4 positive cells had been decreased in the iris at 72 h (Bar = 20 *μ*m). (f) No positive staining was observed in the iris with a nuclear counterstain when under identical experimental conditions when replacing of the primary antibody with normal rabbit IgG at the same concentration (negative control, Bar = 10 *μ*m).

 TLR4 positive cells were brown, which were located in the cellular membrane. TLR4 could not be detected in the iris-ciliary body complex in 0 h group (Figure 4(a)); at 12 h, much TLR4 positive cells were found around blood vessels (Figure 4(b)); the number of TLR4 positive cells significantly increased in the iris and ciliary body of all rats at 24 h (Figure 4(c)) and reached the peak at 48 h (Figure 4(d)). The number of positive cells had reduced at 72 h (Figure 4(e)). There was statistical significance to positive cells overall among these groups (*F* = 46.79, *P* < .05 ANOVA). A small amount of positive cells were also seen in ciliary body. However, no positive cells could be detected in negative control with a nuclear counterstain (Figure 4(f)).

### 3.4. Expression of MyD88

**Figure 5:**
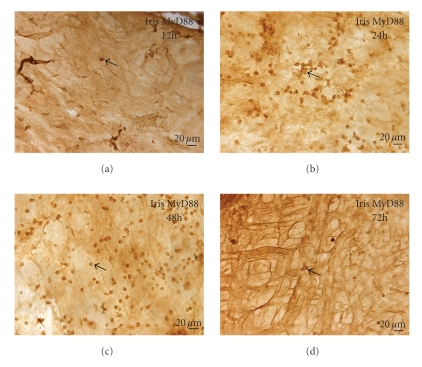
Immunohistochemistry of MyD88 (DAB): (a) The MyD88 positive cells were shown at 12 h. (b) Most of MyD88 expressed in the iris at 24 h. (c) The immunopositive cells were predominantly round-ovoid cells at 48 h. (d) MyD88 positive cells had been decreased in the iris at 72 h (Bar = 20 *μ*m).

MyD88 positive cells were mainly located in the cytoplasm. The trend of changes in MyD88 expression was consistent with the TLR4 (Figure 2(b)). MyD88 could not be detected at 0 h, but positive cells were observed in iris at 12 h (Figure 5(a)) and reached the maximum during 24~48 h (Figures 5(b) and 5(c)). The number of positive cells had reduced at 72 h (Figure 5(d)). No positive cells could be detected in negative control. There was statistical significance to positive cells among these groups (*F* = 54.37, *P* < .05 ANOVA).

### 3.5. Expression of NF-*κ*B p65

**Figure 6:**
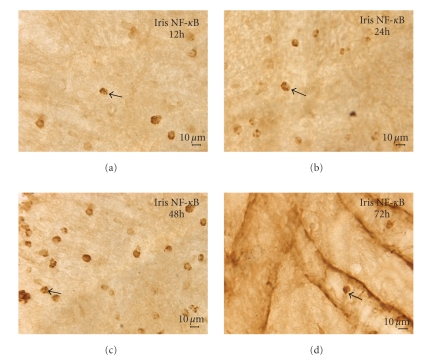
Immunohistochemistry of NF-*κ*B p65 (DAB): (a) The NF-*κ*B p65 positive cells were shown at 12 h. (b) The NF-*κ*B p65 positive cells increased at 24 h. (c) NF-*κ*B p65 located in the cytoplasm or nucleus at 48 h. (d) NF-*κ*B p65 positive cells had been decreased in the iris at 72 h (Bar = 10 *μ*m).

NF-*κ*B p65 could not be found at 0 h group, but NF-*κ*B p65 positive cells were located in the cytoplasm or nucleus at other groups and its expression was gradually increased from 12 h to 48 h. Immunopositive cells were predominantly round-ovoid cells mainly scattering in iris stroma. The number of positive cells had reduced at 72 h. There was statistical significance to positive cells among these groups (*F* = 85.32, *P* < .05, Figures 6(a)–6(d).

## 4. Discussion

Uveitis is a common inflammatory disease that was a potential threaten to visual loss, which mainly affects iris, ciliary body, and choroid [9]. At present, the pathogenic mechanisms of uveitis is not clear. The majority of uveitis may be caused by nonimmune factors, only a small part of the infectious uveitis is due to pathogen invasion. Acute anterior uveitis, especially HLA-B27-associated AAU is a common noninfectious uveitis, but clinical and laboratory research have proven gram negative bacteria such as Klebsiella, Salmonella, Yersinia, and Shigella species can trigger it [10]. TLR-4 is a main receptor that recognizes lipopolysaccharide of gram-negative bacterial cell wall. In our study, TLR4 was not expressed in the normal Wistar rat iris whereas Chang et al. [11] and Brito et al. [12] found that TLR4 positive cells expressed in the normal hunman iris-ciliary body. This may be due to the different subjects and methods applied since TLR4 expression is too low in normal Wistar rat to be detected by iris stretched preparation technology. In our study, we found that TLR4 was expressed in iris-ciliary body after LPS administration. The inflammatory response reached the maximum at 24 h after LPS administration, then the degree of inflammatory response was gradually reduced, but TLR4 positive cells continued to increase until 48 h in the iris. TLR4 expression significantly decreased at 72 h comparing with it at 48 h (*P* < .001). In our study, we had observed that the changes of TLR4 was relative to the degree of anterior segment inflammation, which suggested that LPS-related gram negative bacteria could excessively activate TLR4-mediated innate immunity and adaptive immunity that may resulted in incidence of AAU.

Upon LPS recognition, TLR4 undergoes oligomerization and recruits its downstream adaptors through interactions with the TIR (Toll-interleukin-1 receptor) domains, resulting in inflammatory reaction finally [13]. TLR4 signaling has been divided into MyD88-dependent and MyD88-independent (TRIF-dependent) pathways. Our study found that many MyD88 positive cells were expressed in the iris at 24 h after LPS administration, peaked at 48 h, and then gradually decreased. The curve of MyD88 expression is consistent with TLR4. It showed that TLR4 activated its downstream signaling molecules through a MyD88-dependent pathway conduction in the pathogenesis of AAU. Su et al. [14] reported that MyD88-deficient mice were completely resistant to experimental autoimmune uveitis (EAU) in Th1 mediated autoimmunity response. Taken together, these findings suggest MyD88-dependent pathway plays an essential role in LPS/TLR4 signaling. 

After MyD88 activation, another adaptor protein TRAF6 (TNF receptor-associated factor 6) is critical for the MyD88-dependent pathway. It leads to the phosphorylation of I*κ*B proteins which makes NF-*κ*B/I-*κ*B trimer complex degradation. Subsequencely, NF-*κ*B is activated and transfered into nucleus [13]. In our study, NF-*κ*B p65 positive cells could not be observed in normal Wistar rat iris. At 12 h after LPS immunization, NF-*κ*B p65 positive cells were detected in the cytoplasm and nucleus of iris, reaching the maximum at 48 h, and then had decreased at 72 h. Compared to TLR4 and MyD88, NF-*κ*B p65 reduction is more obvious from 48 h to 72 h, which can better reflect attenuation of inflammation directly. It is consistent with Chi et al. [15] report in that NF-*κ*B p65 was activated in iris-ciliary body after a footpad injection of LPS in the rats. Under the normal circumstances, the p65 subunit of NF-*κ*B binds with its inhibitor I-*κ*B to form I-*κ*B-NF-*κ*Bp50/p65 trimeric complex, which is in the nonactivation state. When the I-*κ*B inhibitory protein degradates, NF-*κ*B antibody recognizes and activates p65 [16]. In addition, Todaro et al. [17] found that NF-*κ*B was highly expressed in Behcet's peripheral blood T cells, which contributes to the regulation of the apoptosis-related factors and death receptors leading to apoptosis resistance in BD T cell subsets. In the research, the reduction of NF-*κ*B p65 may be induced in the down regulation of the peripheral lymphocyte apoptosis by this transduction pathway, which results in regression of inflammation reaction. We found more positive TLR4 and MyD88 cells than NF-*κ*B p65 cells in the same field at 72 h, which may be due to feedback in the pathway. Further studies, including functional studies, are required to identify the roles of nuclear factor in the network of uveitis.

## 5. Conclusions

Our study revealed that the expression of TLR4, MyD88, and NF-*κ*B p65 in the iris changed during EIU. These findings suggest the important role of TLR4 and its associated factors in the pathogenesis of uveitis and will provide some insightful ideas of the mechanism of uveitis.

## Figures and Tables

**Table 1:** Clinical scoring of endotoxin-induced uveitis and density of immunopositive cells in the rat iris.

Time (after LPS injection)	clinical grade (mean ± SD *n* = 10)	TLR4^+^ (Cell/mm^2^, *n* = 10)	MyD88^+^ (Cell/mm^2^ *n* = 10)	NF-*κ*B p65^+^ (Cell/mm^2^ *n* = 10)
0 h	0 ± 0	0 ± 0	0 ± 0	0 ± 0
12 h	2.2 ± 0.4	167.9 ± 61.5	154.3 ± 41.6	220.0 ± 54.6
24 h	4.0 ± 0	780.0 ± 191.9	1040.7 ± 209.2	402.1 ± 55.4
48 h	3.1 ± 0.3	917.9 ± 194.6	1034.6 ± 267.3	807.9 ± 177.2
72 h	1.7 ± 0.5	573.6 ± 113.3	650.8 ± 97.8	150 ± 57.9
